# Waldenström macroglobulinemia with extramedullary involvement at initial diagnosis portends a poorer prognosis

**DOI:** 10.1186/s13045-015-0172-y

**Published:** 2015-06-24

**Authors:** Xin Cao, Qing Ye, Robert Z. Orlowski, Xiaoxiao Wang, Sanam Loghavi, Meifeng Tu, Sheeba K. Thomas, Jatin Shan, Shaoying Li, Muzaffar Qazilbash, C. Cameron Yin, Donna Weber, Roberto N. Miranda, Zijun Y. Xu-Monette, L. Jeffrey Medeiros, Ken H. Young

**Affiliations:** Department of Hematopathology, The University of Texas MD Anderson Cancer Center, 1515 Holcombe Boulevard, Houston, TX 77030 USA; Department of Lymphoma and Myeloma, The University of Texas MD Anderson Cancer Center, Houston, TX USA; Department of Hematology, the Affiliated Hospital of Nantong University, Nantong, China; Department of Stem Cell Transplantation, The University of Texas MD Anderson Cancer Center, Houston, TX USA; The University of Texas School of Medicine, Graduate School of Biomedical Sciences, Houston, TX USA

**Keywords:** Waldenström macroglobulinemia, Lymphoplasmacytic lymphoma, Extramedullary, Prognosis, Treatment

## Abstract

**Background:**

The prognostic importance of extramedullary involvement in patients with Waldenström macroglobulinemia (WM) at diagnosis and treatment options for these patients has not been well evaluated. In this study, we investigated the clinical manifestations, biological features, and effect of first-line therapy on the outcome of WM patients diagnosed with extramedullary involvement (EMWM) vs those with only bone marrow involvement (BMWM).

**Methods:**

We analyzed the clinical data of 312 WM patients diagnosed with EMWM (*n* = 106) and BMWM (*n* = 206) at The University of Texas MD Anderson Cancer Center from 1994 to 2014. EMWM was confirmed by biopsy, positron emission tomography–computed tomography, or magnetic resonance imaging, and clinical laboratory analyses.

**Results:**

Characteristics associated with EMWM were male sex (*P* = 0.027), age younger than 65 years (*P* = 0.048), presence of B symptoms (*P* < 0.001), high serum beta-2 macroglobulin (*P* < 0.001) level, low serum albumin level (*P* = 0.036), and cytogenetic abnormalities (*P* = 0.010). Kaplan-Meier survival analysis results showed that EMWM patients had a significantly shorter median overall survival (*P* < 0.001) and progression-free survival (PFS) (*P* < 0.001) than did BMWM patients. Chemotherapy combined with targeted therapy improved PFS for BMWM patients (*P* = 0.004) but not for EMWM patients. Additionally, initial treatment with rituximab significantly improved the PFS of BMWM patients (*P* = 0.012) but had no effect on EMWM patients. However, EMWM patients treated with nucleoside analogs attained a better PFS than those who did not (*P* = 0.021).

**Conclusions:**

We show that extramedullary involvement at diagnosis is an adverse prognostic factor in WM patients and that first-line therapy with nucleoside analogs improved PFS for patients with EMWM. The study provides unique clinical and treatment observations in subtypes of WM patients.

## Background

Since the initial description of Waldenström macroglobulinemia (WM) in 1944, there have been great advances in the definition and our understanding of this disease [1]. Waldenström macroglobulinemia, characterized by infiltration of the bone marrow (BM) with B-lymphocytes, lymphoplasmacytic cells, and plasma cells, along with detectable serum monoclonal immunoglobulin M (IgM) of any level, is also considered to be a subset, albeit a very large subset, of lymphoplasmacytic lymphoma (LPL) [2]. Approximately 1000–1500 patients are diagnosed with WM per year in the United States [3].

Most of the patients with WM at time of initial diagnosis are with disease restricted to the bone marrow. They may have symptoms including anemia, other peripheral cytopenias, less commonly hyperviscosity syndrome, and paraprotein deposition in various tissues, as a result of tumor cell secretion of paraprotein. They also can have symptoms associated with tumor cells infiltrating lymph nodes or other organs. Although WM is classified as a non-Hodgkin lymphoma, WM involves lymph nodes or other organs less often than other types of non-Hodgkin lymphoma [4]. The unique features of WM are captured in its own, distinct staging system (the International Staging System for WM [ISSWM]), which includes: age, hemoglobin (Hb), platelet count (PLT), beta-2 microglobulin level (β2-MG), and IgM level [5]. Extramedullary involvement by WM at diagnosis has not been included in the 5 prognostic factors of ISSWM, and no report has systematically focused on the treatment of WM patients who have extramedullary involvement.

The wide use of advanced imaging techniques, such as positron emission tomography (PET), magnetic resonance imaging (MRI), and enhanced computed tomography (CT) has facilitated the evaluation of the extent of involvement by WM and the identification of those WM patients diagnosed with extramedullary lesion involvement (EMWM) at time of initial diagnosis. In our clinical practice, a third of WM patients are diagnosed with extramedullary involvement at time of diagnosis. No study has focused systematically on the outcomes of first-line treatment for patients with EMWM and patients with only bone marrow involvement (BMWM). We report our experience regarding the clinical manifestations and biological features of WM associated with EM involvement, as well as the effect of first-line therapy on patient outcomes, in a large population of patients at our institution.

## Results

### Clinical characteristics of patients of WM

A total of 312 patients with WM were identified, with a median age of 62 years (range, 28–87 years) and a male to female ratio of 1.2:1. The ethnic distribution of cases was: 278 white, 14 Hispanic, 13 African-American, and 7 Asian. Eighty-five (27.2 %) patients had B symptoms, 35 (11 %) had neuropathy, 32 (10.3 %) had visual problems, and 25 (8 %) had amyloidosis. According to the IPSSWM, 104 (33.3 %) patients were categorized in the low-risk group (IPSSWM I), 117 (37.5 %) patients in the medium-risk group (IPSSWM II), and 91 (29.2 %) patients in the high-risk group (IPSSWM III). A total of 240 symptomatic patients, who were previously untreated, received therapeutic agents for WM, including alkylating agents (chlorambucil, melphalan, and cyclophosphamide), nucleoside analogs (fludarabine, cladribine), rituximab, alone or combinations of these drugs, and more recently, thalidomide-based regimens, bortezomib, and bendamustine. The remainder of the cohort included 52 patients who were observed (watchful waiting), 11 patients treated in other ways like having Chinese herbs, X only received radiotherapy, and 9 patients either refused treatment or detailed treatment information was not available. With a median follow-up of 66 months (range, 1–292 months), the 5-year estimates of overall survival (OS) and progression-free survival (PFS) were 84 and 49 %, respectively, for all WM patients. Using univariate analysis, we found that factors associated with shorter median OS included: age over 65 years at diagnosis, B symptoms, PLT count <100 × 10^9^/L, LDH >618 IU/L, β2-MG >3 mg/L, albumin (ALB) <3.5 g/dL, and extramedullary involvement (P1 = 0.002, P2 = 0.001, P3 = 0.030, P4 = 0.070, P5 = 0.001, P6 < 0.001, P7 = 0.001).

### Immunophenotypic and cytogenetic findings

Consistent with the WHO classification of WM, most of the immunophenotypic profiles of the WM were negative for CD5, CD10, and CD23 and were positive for CD20, CD19, IgM, and monotypic surface immunoglobulin light chain (k-predominant, 95.2 %). Variable expression of CD5, CD10, and CD23 was found in a subset of patients (CD5^+^ 8.9 %, CD10^+^ 1.8 %, CD23^+^ 1.8 %). Conventional cytogenetic analysis data were available for 261 patients. An abnormal karyotype was observed in 88 (33.7 %) patients. Fourteen patients had isolated loss of the Y chromosome, 37 patients had another single chromosomal abnormality, 22 patients had a complex karyotype (≥3 abnormalities), and the remaining 15 patients had 2 miscellaneous abnormalities. Deletion of 6q was found in 18 patients (deletion was isolated in 3 patients, accompanied by 1 additional abnormality in 4 patients, and was part of a complex karyotype in 11 patients).

### Extramedullary presentation and pathologic pattern of EMWM at diagnosis

**Fig. 1:**
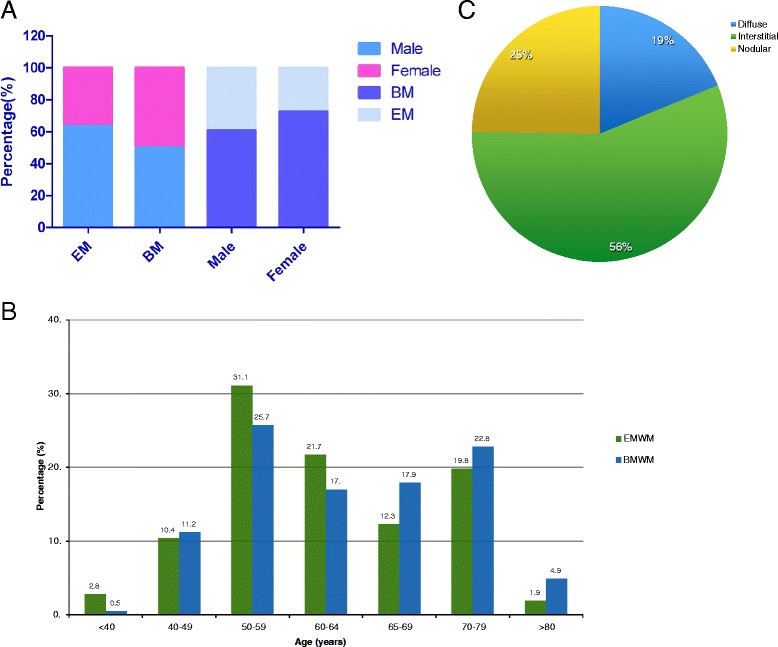
Baseline characteristics of patients diagnosed with Waldenström macroglobulinemia (WM). **a** Gender distribution pattern of patients with WM. **b** Age distribution of EMWM patients and BMWM patients. **c** Pathology pattern of patients with EMWM

**Fig. 2:**
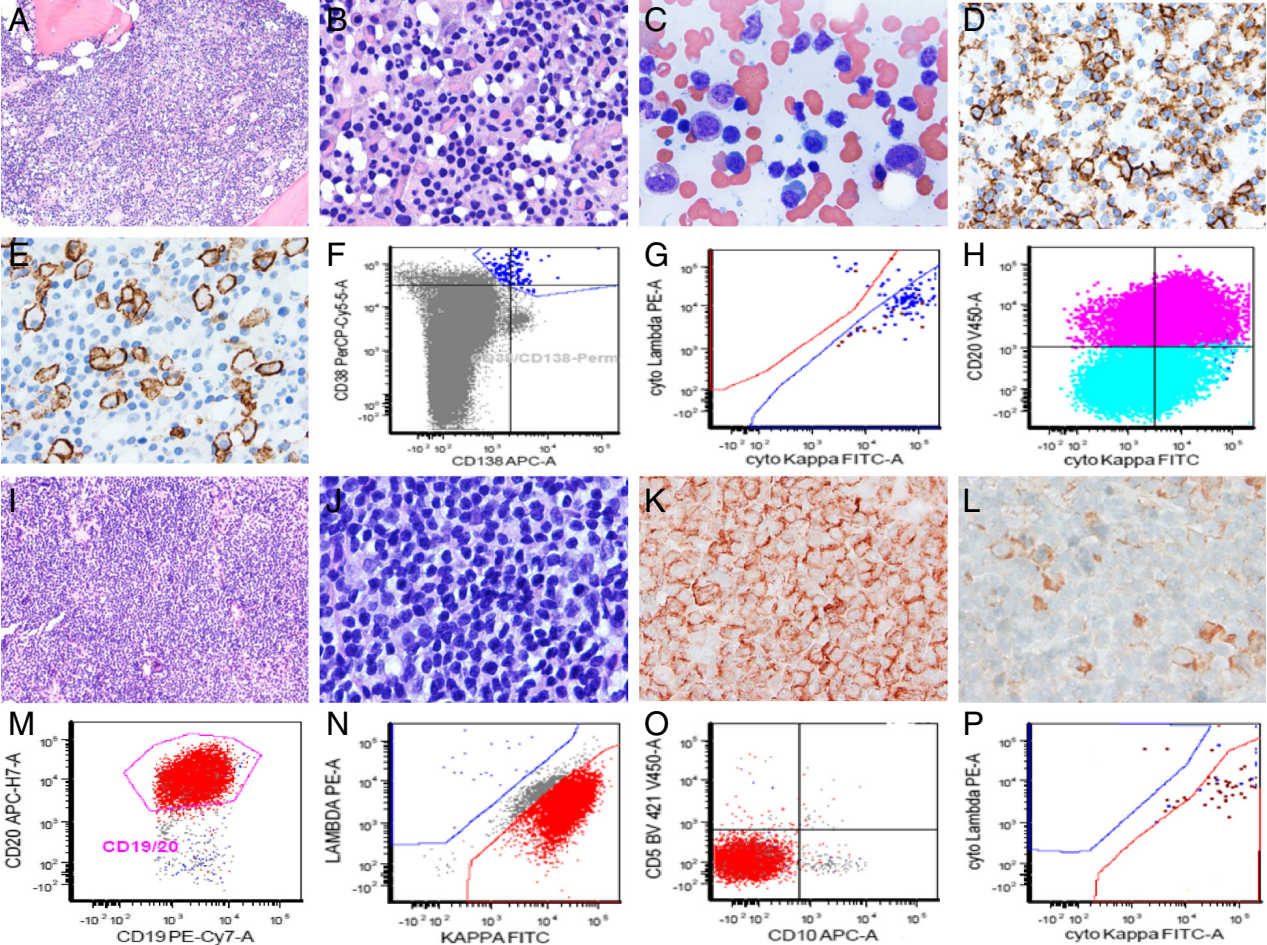
The tissue or bone marrow involvement pattern and immunophenotypic profiling of the EMWM and BMWM patients. **a**, **b** A representative diffuse interstitial pattern of bone marrow biopsy in a patient with BMWM, ×40 and × 80 magnification. **c** Bone marrow aspirate smear showed many abnormal small lymphoid cells admixed with variable plasmacytoid cells and plasma cells, ×80 magnification. **d** CD20 stain on the abnormal small lymphoid cells, ×80 magnification. **e** CD138 stain on the plasmacytoid and plasma cells, ×80 magnification. **f**, **g** Immunophenotypic profiling of CD38 and CD138 positive plasmacytoid and plasma cells with strong monotypic cytoplasmic kappa light chain expression. **h** The abnormal small lymphoid cells were CD20 positive and showed monotypic kappa light chain expression. **i**, **j** A representative diffuse infiltrative pattern of a lymph node biopsy in a patient with EMWM, MYD88 mutation positive, ×40 and × 80 magnification. **k** CD20 stain on abnormal small lymphoid cells, ×80 magnification. **l** CD138 stain on few admixed plasmacytoid and plasma cells, ×80 magnification. **m**, **o** Immunophenotypic profiling of CD19 and CD20 positive small B-cells with kappa light chain restriction and were negative for CD5 and CD10. **p** Identical monotypic kappa light chain expression in the lymphoid cells was also seen in the plasmacytoid and plasma cells

A total of 106 patients were confirmed as having EMWM at time of diagnosis, with a median follow-up time of 63 months; 67 patients (63.4 %) were male while 39 patients were female (36.6 %) (Fig. 1a). Seventy (66 %) patients were less than 65 years old (Fig. 1b), and lymph nodes in the abdomen were the most common extramedullary site (*n* = 71). Other extramedullary sites included axillary or mediastinal lymph nodes (*n* = 34), head/neck lymph nodes (*n* = 24), spleen (*n* = 23), pleura (*n* = 13), abdominal mass (*n* = 8), kidney (*n* = 5), liver (*n* = 2), central nervous system (*n* = 2), muscle (*n* = 2), orbital soft tissue (*n* = 1), maxillary sinus (*n* = 1), and adipose tissue (*n* = 1). Bone marrow involvement of these patients was interstitial in most cases (56 %) (Fig. 1c). The tissue or bone marrow involvement pattern and immunophenotypic profiling of representative EMWM and BMWM patients are shown in Fig. 2. Regardless of BMWM and EMWM, the neoplastic cells commonly exhibit a diffuse infiltrative pattern and are mainly composed of abnormal small lymphoid cells admixed with variable plasmacytoid cells and plasma cells (Fig. 2a–c, i–j). They are negative for CD5, CD10, and CD23 but with strong monotypic immunoglobulin light chain expression (Fig. 2o).

### Different clinical characteristics and survival pattern of EMWM and BMWM group

**Fig. 3:**
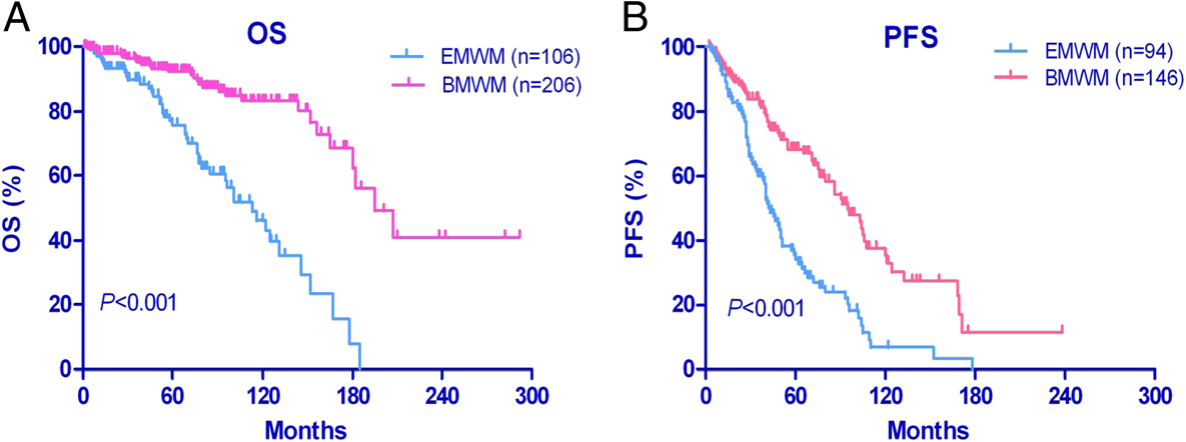
Overall survival (OS) and progression-free survival (PFS) of patients. **a** The Waldenström macroglobulinemia with extramedullary involvement (EMWM) group had shorter OS than did the WM patients with only bone marrow involvement (BMWM) group. **b** In the symptomatic WM patients with standard treatment, the PFS of the EMWM group was also significantly shorter than that of the BMWM group

Table 1 compares the baseline clinical characteristics of patients with EMWM and those with BMWM. Of the EMWM patients (*n* = 106), 67 (63.2 %) were men and 39 (36.8 %) were women. The sex and age pattern of the EMWM and BMWM groups are shown in Fig. 1a, b. The abdomen was the most common site of lymph node infiltration in the EMWM group (*n* = 71). The EMWM group compared with the BMWM group had significantly more male patients (*P* = 0.027) and patients younger than 65 years (*P* = 0.048); a higher rate of B symptoms (*P* < 0.001); a higher frequency of decreased ALB levels (*P* = 0.036); greater rate of increased β2-MG level (*P* < 0.001); and a higher rate of cytogenetic abnormalities (*P* = 0.010). The proportion of IPSSWM III patients was higher in the EMWM group (36.8 %) than in the BMWM group (25.2 %), although this difference was not statistically significant (*P* = 0.064). The EMWM and BMWM groups had similar Hb, PLT, IgM, and LDH levels and a similar percentage of BM involvement. The tissue or bone marrow involvement pattern and immunophenotypic profiling of the EMWM and BMWM patients are shown in Fig. 2a-p. Using Kaplan-Meier survival analysis, the EMWM patients had remarkably shorter median OS (P < 0.001; Fig. 3a) and PFS (P < 0.001; Fig. 3b) than did BMWM patients. On univariate analysis, we found that age older than 65 years and low ALB level were poor prognostic factors for EMWM patients (Table 2). Multivariate analysis revealed that EMWM patients with low ALB levels had shorter median OS than EMWM patients with normal ALB levels. On both univariate and multivariate analysis, EMWM patients with cytogenetic abnormalities had a shorter median OS and PFS than those with a normal karyotype. The result of multivariate analysis in the EMWM was different from that of WM patients as a whole (Table 3).

**Table 1:** Different clinical manifestations and biological features between Waldenström macroglobulinemia (WM) patients with only bone marrow involvement (BMWM) and WM with extramedullary involvement (EMWM)

Characteristic	BMWM	EMWM	*P* ^a^
	*n* (%)	*n* (%)	
Age			*0.048*
≥ 65 years	94 (45.6)	36 (34.0)	
< 65 years	112 (54.4)	70 (66.0)	
Sex			*0.027*
Male	103 (50.0)	67 (63.4)	
Female	103 (50.0)	39 (36.6)	
Hb			0.240
≤ 11.5 (g/dL)	112 (54.4)	65 (61.3)	
> 11.5 (g/dL)	94 (45.6)	41 (38.7)	
PLT			0.525
≤ 100 (×109/L)	44 (21.4)	26 (24.5)	
> 100 (×109/L)	162 (78.6)	80 (75.5)	
IgM			*0.726*
> 70 (g/L)	20 (9.7)	9 (8.5)	
≤ 70 (g/L)	186 (90.3)	97 (91.5)	
β2-MG			*<0.001*
> 3 (mg/L)	75 (36.4)	71 (64.3)	
≤ 3 (mg/L)	131 (63.6)	35 (35.7)	
LDH (*n* = 304)			0.906
> 618 (IU/L)	24 (12.0)	12 (11.5)	
≤ 618 (IU/L)	176 (88.0)	92 (88.5)	
B symptoms			*<0.001*
Present	41 (19.9)	44 (41.5)	
Absent	165 (80.1)	62 (58.5)	
ALB (*n* = 307)			*0.036*
< 3.5 g/dL	23 (11.3)	21 (20.2)	
≥ 3.5 g/dL	180 (88.7)	83 (79.8)	
IPSSWM stage			0.064
I	69 (33.5)	35 (33.0)	
II	85 (41.3)	32 (30.2)	
III	52 (25.2)	39 (36.8)	
Cytogenetic findings (*n* = 261)			*0.010*
Abnormal	48 (28.2)	40 (44.0)	
Normal	122 (71.8)	51 (56.0)	
BM involvement	44.52 ± 1.79	46.50 ± 2.49	0.529
(mean ± SD, %)			

*Hb* hemoglobin, *PLT* platelet, *IgM* immunoglobulin M, *β2-MG* beta-2 macroglobulin level, *LDH* lactate dehydrogenase, *ALB* albumin, *IPSSWM*, International Prognostic Scoring System for WM

^a^ *P* value by chi-square and Mann–Whitney U tests; statistically significant values appear in italics

**Table 2:** Univariate analysis of overall survival (OS) and progression-free survival (PFS) for patients diagnosed with Waldenström macroglobulinemia (WM) with extramedullary involvement (EMWM)

	EMWM						WM					
	OS			PFS			OS			PFS		
	HR	95 % CI	*P* ^a^	HR	95 % CI	*P* ^a^	HR	95 % CI	*P* ^a^	HR	95 % CI	*P* ^a^
Age ≥65 years	1.90	1.02–3.54	*0.044*	1.04	0.63–1.73	0.876	2.09	1.32–3.32	*0.002*	1.09	0.77–1.56	0.598
Sex (male)	0.76	0.41–1.39	0.372	0.74	0.45–1.22	0.239	1.06	0.59–1.50	0.799	0.99	0.70–1.39	0.955
Race (white)	0.72	0.29–1.73	0.465	0.54	0.29–1.02	0.057	0.66	0.31–1.40	0.281	0.59	0.35–0.98	*0.042*
EM-positive	-	-	-	-	-	-	4.17	2.56–6.79	*<0.001*	2.38	1.69–3.34	*<0.001*
IPSSWM (I, II vs III)	1.37	0.95–1.97	0.097	1.08	0.81–1.45	0.588	1.85	1.35–2.50	*<0.001*	1.18	0.96–1.47	0.124
B symptom (+)	1.38	0.75–2.52	0.296	1.05	0.65–1.68	0.842	2.36	1.44–3.85	*0.001*	1.26	0.88–1.81	0.209
WBC <4 × 10^12^/L	1.39	0.69–2.82	0.360	1.41	0.80–2.47	0.234	1.09	0.51–1.65	0.776	1.19	0.79–1.80	0.395
Hb ≤11.5 g/L	1.26	0.68–2.34	0.460	0.97	0.59–1.57	0.886	1.53	0.46–2.45	*0.076*	0.99	0.70–1.40	0.958
PLT <100 × 10^9^/L	1.63	0.88–3.06	0.123	1.37	0.81–2.32	0.243	1.78	1.06–2.99	*0.030*	1.59	1.08–2.36	*0.019*
IgM >7000 mg/L	0.24	0.03–1.74	0.158	0.40	0.14–1.10	0.075	0.35	0.08–1.43	0.143	0.51	0.26–1.00	0.052
β2-MG >3 mg/L	1.33	0.68–2.57	0.395	1.15	0.70–1.90	0.598	2.18	1.35–3.50	*0.001*	1.38	0.98–1.94	0.063
ALB <3.5 g/dL	2.12	1.12–4.00	*0.020*	1.21	0.69–2.13	0.501	2.64	1.61–4.35	*<0.001*	1.53	1.00–2.32	*0.049*
LDH >618 IU/L	1.18	0.41–3.37	0.757	1.49	0.75–2.93	0.250	1.78	0.95–3.31	*0.070*	2.02	1.29–3.15	*0.002*
Cytogenetic (abnormal)	1.08	0.57–2.03	0.823	1.71	1.03–2.83	*0.039*	1.37	0.83–2.23	0.208	1.77	1.23–2.55	*0.002*
BM involvement	0.99	0.97–1.00	0.093	0.99	0.98–1.00	0.172	1.00	0.99–1.01	0.666	1.00	0.99–1.00	0.417

*CI* confidence interval, *HR* hazard ratio, *EM* extramedullary, *IPSSWM* International Prognostic Scoring System for WM, *WBC* white blood cell account, *Hb* hemoglobin, *PLT* platelet, *IgM* immunoglobulin M, *β2-MG* beta-2 microglobulin level, *ALB* albumin, *LDH* lactate dehydrogenase, *BM* bone marrow

^a^ *P* value by Cox proportional hazards regression model; statistically significant values appear in italics

**Table 3:** Multivariate analysis of overall survival (OS) and progression-free survival (PFS) for patients diagnosed with Waldenström macroglobulinemia (WM) with extramedullary involvement (EMWM)

	OS				PFS		
Variable	HR	95 % CI	*P* ^a^	Variable	HR	95 % CI	*P* ^a^
EMWM							
Age ≥65 years	1.41	0.67–2.97	0.367	Race (white)	0.50	0.25–1.00	0.051
IPSSWM (I, II vs III)	1.41	0.90–2.21	0.138	IgM >7000 mg/L	0.42	0.15–1.16	0.094
ALB <3.5 g/dL	2.09	1.07–4.07	*0.031*	Cytogenetic (abnormal)	1.86	1.09–3.17	*0.022*
BM involvement	0.99	0.98–1.00	0.080	-	-	-	-
WM							
Age ≥65 years	3.13	1.85–5.29	*<0.001*	Race (white)	0.57	0.32–1.05	0.070
Hb ≤11.5 g/L	1.12	0.65–1.93	0.685	IgM >7000 mg/L	0.49	0.23–1.04	0.064
B symptom–positive	1.25	0.72–2.17	0.428	Cytogenetic (abnormal)	1.78	1.21–2.63	*0.003*
EM–positive	4.28	2.42–7.55	*<0.001*	EM–positive	2.06	1.39–3.06	*<0.001*
PLT <100 × 10^9^/L	1.14	0.63–2.05	0.663	PLT <100 × 10^9^/L	0.98	0.60–1.59	0.935
ALB <3.5 g/dL	2.10	1.24–3.54	*0.006*	ALB <3.5 g/dL	1.12	0.69–1.80	0.648
LDH >618 IU/L	2.00	0.98–4.06	0.054	LDH >618 IU/L	1.47	0.87–2.51	0.153
β2-MG >3 mg/L	1.36	0.78–2.38	0.277	β2-MG >3 mg/L	1.42	0.92–2.17	0.111

*CI* confidence interval, *HR* hazard ratio, *IPSSWM* International Prognostic Scoring System for WM, *IgM* immunoglobulin M, *ALB* albumin, *BM* bone marrow, *Hb* hemoglobin, *PLT* platelet, *LDH* lactate dehydrogenase, *β2-MG* beta-2 microglobulin level

^a^ *P* value by Cox proportional hazards regression model; statistically significant values appear in italics

### Outcomes of initial treatments

**Fig. 4:**
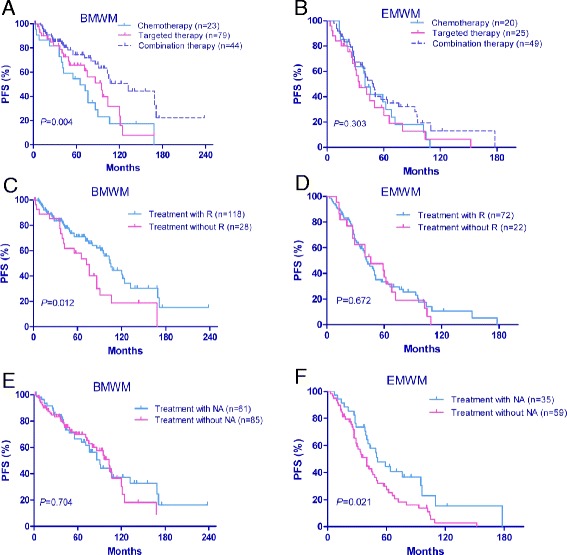
Progression-free survival (PFS) of symptomatic Waldenström macroglobulinemia (WM) patients according to different initial treatments. **a** Chemotherapy combined with targeted therapy significantly improved the PFS of patients with WM and only bone marrow involvement (BMWM) but had no effect on patients diagnosed with WM with extramedullary involvement (EMWM) (**b**). **c** BMWM patients who received treatment with rituximab had better PFS than those treated without rituximab. **d** Rituximab did not improve PFS in patients with EMWM. **e** Nucleoside analogs did not affect the outcome of patients with BMWM but improved the PFS of patients with EMWM (**f**)

The general information for the first-line treatment of WM patients is summarized in Table 4. Eight of the EMWM patients and 44 of the BMWM patients chose to be observed. Another 20 patients were treated with alternative medicine or radiation, refused treatment, or did not have detailed treatment information available in the medical records. These patients were excluded from the treatment analysis. The treatment analysis thus included 240 WM patients who had systemic therapy: 146 BMWM patients and 94 EMWM patients. We first categorized these 240 patients into the following 3 treatment groups: (1) chemotherapy group, which included cytotoxic drugs such as alkylating agents (chlorambucil and cyclophosphamide) and nucleoside analogs, NAs (fludarabine and cladribine); (2) targeted therapy group, which included regimens containing the monoclonal antibody rituximab and the proteasome inhibitor bortezomib, with or without dexamethasone, but without cytotoxic chemotherapy; and (3) combination group, which included both cytotoxic chemotherapy and biological agents. The major response rates to first-line therapy (of any kind) were 90 and 92 % in the EMWM and BMWM groups, respectively. BMWM patients treated with a combination of targeted agents and chemotherapy had significantly longer median PFS (*P* = 0.004) than did BMWM patients in the other 2 treatment groups, but combination treatment had no significant effect on the EMWM group (Fig. 4a, b). Similar results were shown in the analysis of patients treated with rituximab and without rituximab. Initial treatment including rituximab improved the PFS of BMWM patients (*P* = 0.012) (Fig. 4c), however, there was no difference in the median OS or PFS on EMWM patients treated with rituximab (Fig. 4d). We found that EMWM patients treated with NA had longer median PFS (*P* = 0.021) than those patients not treated with NA (*P* = 0.821), but NA regimens did not affect PFS significantly in the BMWM group (Fig. 4d, e). No remarkable differences in OS were observed for the different treatment groups.

**Table 4:** Initial therapy used to treat symptomatic patients diagnosed with Waldenström macroglobulinemia (WM) with only bone marrow involvement (BMWM) and those with extramedullary involvement (EMWM)

Type of therapy	EMWM (*n* = 106)	BMWM (*n* = 206)
	*n* (%)	*n* (%)
Treated for WM		
Yes	94 (88.67)	146 (70.87)
Observation only	8 (7.55)	44 (21.36)
Alternative medicine	2 (1.89)	9 (4.37)
Missing data	2 (1.89)	7 (3.40)
Initial treatment		
Chemotherapy	20 (18.87)	23 (11.17)
Targeted therapy	25 (23.58)	79 (38.35)
Combination	49 (46.23)	44 (21.36)
Initial treatment with R		
Yes	72 (67.92)	118 (57.28)
No	22 (20.75)	28 (13.59)
Initial treatment with NA		
Yes	35 (33.02)	61 (29.61)
No	59 (55.67)	85 (41.26)

*R* rituximab, *NA* nucleoside analogs

### Secondary hematologic neoplasms

During the follow-up period, 11 WM patients developed secondary hematologic neoplasms: 6 (5.7 %) patients in the EMWM group, including 2 with myeloproliferative neoplasms, 1 with plasma cell myeloma, 2 with diffuse large B-cell lymphoma, and 1 with chronic myelogenous leukemia; and 5 patients in the BMWM group (2.4 %), including 2 with chronic myelogenous leukemia, 2 with diffuse large B-cell lymphoma, and 1 with myeloproliferative neoplasm.

## Discussion

In this study, we have focused on the clinical features and outcomes of EMWM in a large group of WM patients. Lymphadenopathy and organomegaly have been reported in about 20–25 % of patients with WM [6–9]. Other studies have focused mostly on clinical or pathologic aspects of extramedullary disease in WM patients or have excluded some extranodal sites from analysis. In one study, the authors reported that organomegaly was associated with a poorer prognosis [7]. Therefore, the prognostic importance of extramedullary involvement in WM patients has been inadequately addressed. In recent years, advances in imaging modalities and the wide use of these technologies facilitate the detection of EMWM at diagnosis. This study is the first to evaluate systematically the manifestations, treatment, and outcome of extramedullary involvement in WM patients.

Overall, the rate of EMWM at time of diagnosis in this study was 34 %, a little higher than previous reports with increased use of PET-CT. Extramedullary disease has been reported in lymph nodes, the spleen, soft tissue, and specific organs such as the lungs [10], intestines [11], and the central nervous system [12]. In this patient cohort, the most common extramedullary sites were lymph nodes, especially in the abdomen and pelvis, and the spleen. Pleural involvement was observed in 12 % of EMWM patients, and other involved sites included the central nervous system, soft tissue, liver, kidney, mucosa, and intestines, most of the sites that have been previously reported.

The median follow-up for the whole group of WM patients was 66 months, with 5 year-estimates of OS and PFS of 84 and 49 %, respectively. EMWM patients had a significantly shorter median OS and PFS than did BMWM patients (Fig. 3). In the univariate analysis of the whole group, we found that adverse prognostic factors included age of 65 years or older, B symptoms, PLT count <100 × 10^9^/L, high LDH levels, high β2-MG levels, low ALB levels, high ISSWM status, and extramedullary involvement. Compared with BMWM patients, EMWM patients had a higher frequency of B symptoms (*P* < 0.001), high β2-MG levels (*P* < 0.001), decreased ALB levels (*P* = 0.036), and a higher frequency of cytogenetic abnormalities (*P* = 0.010). All of these factors were confirmed to be associated with either shorter median OS or PFS on univariate analysis and also have been reported in previous studies [13].

Male gender was associated with EMWM, and the age distribution of EMWM patients was younger than that of the BMWM patients (*P* = 0.048). Considering shorter life expectancy of elderly people and the association between younger age and extramedullary disease, we suspect that younger than 65 years old is a poor prognostic factor for WM patients.

EMWM patients were more likely to have stage III disease than BMWM patients, which is consistent with a widely accepted international risk stratification system [14]. Yet, IPSSWM stage was not significantly associated with a different prognosis for EMWM patients. Whereas IPSSWM staging showed clear stratification for the whole WM group, we suspect the reason for this phenomenon was the interaction of extramedullary involvement with other factors. Identifying new prognostic factors or refining the scoring system to predict prognostic of WM patients with extramedullary disease would be valuable.

Cytogenetic studies were conducted in most of the patients and showed that significantly more EMWM patients had chromosomal abnormalities than did BMWM patients (*P* = 0.010). Additionally, both univariate analysis and multivariate analysis showed that cytogenetic abnormalities were associated with shorter median PFS, consistent with previous report [15]. However, the abnormal chromosome result by conventional cytogenetic analysis was heterogeneous and test for *MYD88* or *CXCR4* mutation was not routinely performed in these patients. Recent studies have shown that *CXCR4* mutations and WHIM (warts, hypogammaglobulinemia, infections, and myelokathexis syndrome) mutations enhance tumor dissemination and extramedullary involvement within xenograft mouse models of WM [16, 17]. CXCR4, a chemokine receptor that regulates cell trafficking and dissemination, may potentially be a factor in the mechanism of extramedullary involvement in patients with WM. Further studies to detect *MYD88* and *CXCR4* mutations and explore new molecular markers in patients with EMWM may be helpful in clinical practice.

According to current National Comprehensive Cancer Network recommendations [18], WM patients with symptoms related to hyperviscosity syndrome, neuropathy, organomegaly, amyloidosis, cold agglutinin disease, cryoglobulinemia, associated cytopenias, and bulky lymphadenopathy should be treated. Owing to a lack of consensus on the best treatment for WM patients and to the availability of new drugs over the period studied, patients in our study received several types of therapy. Newer biological agents, such as rituximab and bortezomib, have been shown to improve the outcome of WM patients. Consistent with these reports, we found that BMWM patients treated with both biological agents and chemotherapy had significantly better median PFS than patients treated with either chemotherapy or biological agents alone, but combination therapy did not affect PFS in the EMWM group (Fig. 4). The therapeutic benefit of rituximab for WM has been well documented; it can be used as single agent or as a combination with other agents [19, 20]. We also found that BMWM patients treated with rituximab had significantly longer median PFS (*P* = 0.012) and slightly longer median OS (*P* = 0.064) than did BMWM patients who were not treated with rituximab. However, rituximab did not affect survival in EMWM patients.

Conversely, patients in the EMWM group treated with nucleoside analog regimens had significantly better PFS than those not treated with. Nucleoside analogs operate as antimetabolites by inhibiting DNA synthesis and inducing DNA strand breaks. Earlier studies have showed that the NAs, fludarabine and cladribine, either alone or in combination with alkylating agents and rituximab, produced greater response rates and prolonged survival in WM patients [21–23]. In a multicenter prospective study of 43 previously untreated WM patients, the combination of fludarabine, cyclophosphamide, and rituximab showed a satisfactory overall response rate [24]. On the other hand, another study reported an increased risk of disease transformation and the development of myelodysplastic syndrome and secondary acute myelogenous leukemia in WM patients treated with nucleoside analog regimens [25]. Per the recommendations of the National Comprehensive Cancer Network and IWWM-7 [26], the use of nucleoside analogs in young patients who may be potential stem cell transplant candidates should be limited. In this study, 11 WM patients had second hematologic tumor (6 with EMWM and 5 with BMWM). Of these 11 patients, 5 had been treated with nucleoside analogs; 3 of these 5 also received alkylating agents. Another 3 patients had been treated with alkylating agents without nucleoside analogs. Nine of these 11 patients underwent cytogenetic analysis and 7 had of chromosome abnormalities. Our data showed that nucleoside analog regimens did not affect OS or PFS in the BMWM group, therefore, it is insufficient to make a conclusion that nucleoside analog use is a risk factor.

## Conclusions

In conclusion, our data show that extramedullary involvement at diagnosis is an adverse prognostic factor for WM patients. EMWM patients treated with nucleoside analogs had longer median PFS than did patients not treated with nucleoside analogs. Given the conflicting reports about the relationship between NA and malignant transformation risk as well as the limitations of the IPSSWM in predicting the prognosis of patients with EMWM, it is helpful to evaluate new prognostic factors, such as a modified prognostic scoring system or *CXCR4* mutations to improve the rationale for therapeutic decisions.

## Materials and methods

The data for this study were gathered from the electronic medical records of patients with WM seen at the University of Texas MD Anderson Cancer Center, Houston, TX, between 1994 and 2014. Patients without sufficient clinical data and radiographic were excluded. We retrospectively analyzed data on patients with EMWM and patients with bone marrow involvement only (BMWM). Extramedullary disease was detected by PET, MRI, or enhanced CT scans. In some patients, the diagnosis was further confirmed by tissue biopsy. This study was approved by the Institutional Review Board at the University of Texas MD Anderson Cancer Center, Houston, TX, in keeping with institutional, federal, and Helsinki Declaration requirements.

All diagnostic materials were reevaluated for this study to confirm the WM diagnosis according to the World Health Organization (WHO) classification of lymphoid malignancies [2]. All the patients that were diagnosed with WM have bone marrow involvement with lymphoplasmacytoid cell (composed of lymphocytes, plasmacytoid cells, and plasma cells) and show serum IgM paraprotein of variable concentration. In addition, they do not fulfill the criteria for any other B-cell non-Hodgkin lymphomas. To reach accurate diagnosis for LPL/WM patients in our center, series of diagnostic approaches have been used in each of the patients including BM or tissue biopsy, CT-PET, molecular and genetic assay, flow cytometry, and importantly clinical follow-up of treatment response during the disease course. Based on the most recent 2016 WHO Classification Blueprint, MYD88 mutation is present in greater than 95 % of LPL/WM patients. Molecular and genetic studies including MYD88 mutation have been performed in challenging patients to confirm the diagnosis. MYD88 mutation has been used routinely in our center for any possible LPL/WM patients at the current time. As this study is a retrospective analysis, 9 original and 15 untreated follow-up LPL/WM patients were analyzed. MYD88 mutation was identified in most of these patients (22/24, 92 %), which is consistent with reported frequency, supporting that the original diagnosis and classification are likely accurate in majority of the patients. Baseline data were extracted from the clinical databases and medical records. Follow-up information was gathered from each visit record. All patients were human immunodeficiency virus negative.

We collected the following baseline characteristics: age and sex; Hb, PLT count, β2-MG, serum IgM, lactate dehydrogenase (LDH), albumin (ALB), and cytogenetic results. Initial treatment records were also obtained. The IPSSWM score was calculated from these data. Outcomes of patients were evaluated using the criteria established at the Second International Workshop on WM (IWWM) [6]. Data were collected until the time of last follow-up. Because of the typically indolent clinical course of WM, patients usually had repeated remissions and relapses. Therefore, we mainly studied patients’ treatment until the time of first disease progression. For patients who were switched to a more intensive regimen for reasons other than progression of disease were categorized into the group of more intensive regimen for the purposes of this analysis. Reasons other than disease progression leading to a change of therapy included unsatisfactory results from the patient’s perspective, differing opinions of the physicians, or for the purpose of collecting stem cells.

Patient characteristics were summarized using descriptive statistics. Comparisons between two groups were performed with the chi-square and the Mann–Whitney tests. PFS was calculated from the date of diagnosis to the date of first progression, recurrence, or death. OS was calculated from the date of diagnosis to the date of death of any cause or the last follow-up date for censored cases. OS and PFS curves were generated using the Kaplan-Meier method, and differences between groups were compared using the log-rank test. Univariate and multivariate analyses were performed with a Cox proportional hazards regression model. *P* values less than 0.05 were considered statistically significant. All statistical analyses were performed with SPSS software (version 21.0; IBM, Armonk, NY).

## References

[1] Kristinsson SY, Eloranta S, Dickman PW, Andersson TM, Turesson I, Landgren O (2013). Patterns of survival in lymphoplasmacytic lymphoma/Waldenström macroglobulinemia: a population-based study of 1,555 patients diagnosed in Sweden from 1980 to 2005. Am J Hematol.

[2] Swerdlow SH (2008). International Agency for Research on Cancer, and World Health Organization. WHO classification of tumours of haematopoietic and lymphoid tissues, World Health Organization classification of tumours. 4th ed.

[3] Sekhar J, Sanfilippo K, Zhang Q, Trinkaus K, Vij R, Morgensztern D (2012). Waldenström macroglobulinemia: a surveillance, epidemiology, and end results database review from 1988 to 2005. Leuk Lymphoma.

[4] Gertz MA (2005). Waldenström macroglobulinemia: a review of therapy. Am J Hematol.

[5] Gertz MA (2013). Waldenström macroglobulinemia: 2013 update on diagnosis, risk stratification, and management. Am J Hematol.

[6] Owen RG, Kyle RA, Stone MJ, Rawstron AC, Leblond V, Merlini G (2013). Response assessment in Waldenström macroglobulinaemia: update from the VIth International Workshop. Br J Haematol.

[7] Ghobrial IM, Fonseca R, Gertz MA, Plevak MF, Larson DR, Therneau TM (2006). Prognostic model for disease-specific and overall mortality in newly diagnosed symptomatic patients with Waldenström macroglobulinaemia. Br J Haematol.

[8] Lin P, Bueso-Ramos C, Wilson CS, Mansoor A, Medeiros LJ (2003). Waldenström macroglobulinemia involving extramedullary sites: morphologic and immunophenotypic findings in 44 patients. Am J Surg Pathol.

[9] Banwait R, Aljawai Y, Cappuccio J, McDiarmid S, Morgan EA, Leblebjian H (2015). Extramedullary Waldenström macroglobulinemia. Am J Hematol.

[10] Monnet CM, Favrolt N, Bastie JN, Chrétien ML, Benoit F, Rossi C (2014). A rare cause of pulmonary opacities: lung localization of Waldenström’s macroglobulinemia. Rev Mal Respir.

[11] Salamone F, Catanzaro R, Mangiameli A, Castellino P, Puzzo L, Magnano A (2008). Clinical challenges and images in GI. Intestinal Waldenström macroglobulinemia. Gastroenterology..

[12] Ly KI, Fintelmann F, Forghani R, Schaefer PW, Hochberg EP, Hochberg FH (2011). Novel diagnostic approaches in Bing-Neel syndrome. Clin Lymphoma Myeloma Leuk.

[13] Kristinsson SY, Björkholm M, Goldin LR, McMaster ML, Turesson I, Landgren O (2008). Risk of lymphoproliferative disorders among first-degree relatives of lymphoplasmacytic lymphoma/Waldenström macroglobulinemia patients: a population-based study in Sweden. Blood.

[14] Morel P, Merlini G (2012). Risk stratification in Waldenström macroglobulinemia. Expert Rev Hematol.

[15] Mansoor A, Medeiros LJ, Weber DM, Alexanian R, Hayes K, Jones D (2001). Cytogenetic findings in lymphoplasmacytic lymphoma/Waldenström macroglobulinemia. Chromosomal abnormalities are associated with the polymorphous subtype and an aggressive clinical course. Am J Clin Pathol.

[16] Roccaro AM, Sacco A, Jimenez C, Maiso P, Moschetta M, Mishima Y (2014). C1013G/CXCR4 acts as a driver mutation of tumor progression and modulator of drug resistance in lymphoplasmacytic lymphoma. Blood.

[17] Treon SP, Cao Y, Xu L, Yang G, Liu X, Hunter ZR (2014). Somatic mutations in *MYD88* and *CXCR4* are determinants of clinical presentation and overall survival in Waldenström macroglobulinemia. Blood.

[18] Anderson KC, Alsina M, Bensinger W, et al. NCCN (National Comprehensive Cancer Network). Waldenström’s macroglobulinemia/lymphoplasmacytic lymphoma, version 1.2015. http://www.nccn.org/professionals/physician_gls/f_guidelines_nojava.asp. Accessed 20 April 2015.

[19] Tam CS, Wolf M, Prince HM, Januszewicz EH, Westerman D, Lin KI (2006). Fludarabine, cyclophosphamide, and rituximab for the treatment of patients with chronic lymphocytic leukemia or indolent non-Hodgkin lymphoma. Cancer.

[20] Dimopoulos MA, Anagnostopoulos A, Kyrtsonis MC, Zervas K, Tsatalas C, Kokkinis G (2007). Primary treatment of Waldenström macroglobulinemia with dexamethasone, rituximab, and cyclophosphamide. J Clin Oncol.

[21] Tedeschi A, Ricci F, Goldaniga MC, Benevolo G, Varettoni M, Motta M (2013). Fludarabine, cyclophosphamide, and rituximab in salvage therapy of Waldenström’s macroglobulinemia. Clin Lymphoma Myeloma Leuk.

[22] Leblond V, Johnson S, Chevret S, Copplestone A, Rule S, Tournilhac O (2013). Results of a randomized trial of chlorambucil versus fludarabine for patients with untreated Waldenström macroglobulinemia, marginal zone lymphoma, or lymphoplasmacytic lymphoma. J Clin Oncol.

[23] Laszlo D, Andreola G, Rigacci L, Copplestone A, Rule S, Tournilhac O (2010). Rituximab and subcutaneous 2-chloro-2′-deoxyadenosine combination treatment for patients with Waldenström macroglobulinemia: clinical and biologic results of a phase II multicenter study. J Clin Oncol.

[24] Peinert S, Tam CS, Prince HM, Scarlett J, Wolf MM, Januszewicz EH (2010). Fludarabine based combinations are highly effective as first-line or salvage treatment in patients with Waldenström macroglobulinemia. Leuk Lymphoma.

[25] Leleu X, Soumerai J, Roccaro A, Hatjiharissi E, Hunter ZR, Manning R (2009). Increased incidence of transformation and myelodysplasia/acute leukemia in patients with Waldenström macroglobulinemia treated with nucleoside analogs. J Clin Oncol.

[26] Dimopoulos MA, Kastritis E, Owen RG, Kyle RA, Landgren O, Morra E (2014). Treatment recommendations for patients with Waldenström macroglobulinemia (WM) and related disorders: IWWM-7 consensus. Blood.

